# Simultaneous real-time analysis of tear film optical quality dynamics and functional visual acuity in dry eye disease

**DOI:** 10.1186/s40662-023-00333-6

**Published:** 2023-04-02

**Authors:** An-Peng Pan, Yunjing Ma, Ruilin Hu, Xuejiao Cao, Yifen Wu, Kaijing Zhou, Ruixue Tu, Xu Shao, Shihao Chen, A-Yong Yu

**Affiliations:** National Clinical Research Center for Ocular Diseases, Eye Hospital, Wenzhou Medical University, 270 Xueyuan West Road, Wenzhou, 325027 Zhejiang China

**Keywords:** Dry eye disease, Functional visual acuity, Tear film optical quality, Simultaneous real-time base

## Abstract

**Background:**

To assess the effect of tear film instability in dry eye disease (DED) by measuring visual performance and tear film optical quality in a simultaneous real-time analysis system.

**Methods:**

Thirty-seven DED participants and 20 normal controls were recruited. A simultaneous real-time analysis system was developed by adding a functional visual acuity (FVA) channel to a double-pass system. Repeated measurements of FVA and objective scatter index (OSI) were performed simultaneously with this system under blink suppression condition for 20 s. Patient-reported symptoms was evaluated using the Ocular Surface Disease Index (OSDI) questionnaire. Mean FVA, mean OSI, and visual acuity break-up time were defined. The OSI maintenance ratio was calculated as an evaluation index to assess the difference between dynamic OSI changes and baseline OSI. The visual maintenance ratio was also calculated in the same way.

**Results:**

Moderate correlations were noted between mean OSI and FVA-related parameters (mean FVA, visual maintenance ratio, visual acuity break-up time: 0.53, − 0.56, − 0.53, respectively, *P* < 0.01 for all). Moderate to high correlations were noted between OSI maintenance ratio and FVA-related parameters (mean FVA, visual maintenance ratio, visual acuity break-up time: − 0.62, 0.71, 0.64, respectively, all *P* < 0.01). The metrics derived from the simultaneous real-time analysis system were moderately correlated with the patient-reported symptoms and the visual acuity break-up time possessed the highest correlation coefficients with OSDI total, ocular symptoms, and vision-related function (− 0.64, − 0.63, − 0.62, respectively, *P* < 0.01). The OSI-maintenance ratio alone appeared to exhibit the best performance of the metrics for the detection of DED with sensitivity of 95.0% and specificity of 83.8% and the combinations of FVA parameters and OSI parameters were valid and can further improve the discriminating abilities.

**Conclusions:**

OSI-related metrics were found to be potential indicators for assessing and diagnosing DED which correlated with both subjective visual performance and patient-reported symptoms; the FVA-related metrics were quantifiable indicators for evaluating visual acuity decline in DED.

*Trial registration number:* Chinese Clinical Trial Registry, ChiCTR2100051650. Registered 29 September 2021, https://www.chictr.org.cn/showproj.aspx?proj=134612

**Supplementary Information:**

The online version contains supplementary material available at 10.1186/s40662-023-00333-6.

## Background

The high incidence of dry eye disease (DED) is often accompanied by significant socioeconomic impacts, including loss of societal productivity as dry eye symptoms interfere with daily life and work, and decreased quality of life caused by various dry eye symptoms [1–5]. The Dry Eye Workshop II defines DED as a multi-factorial ocular surface disease characterized by a loss of tear film homeostasis [6]. Tear film instability is considered one of the core pathophysiological changes affecting homeostasis. This is manifested by decreased tear volume, accelerated spontaneous tear film rupture, and increased tear evaporation from the ocular surface [7].

A uniform and stable precorneal tear film is essential for maintaining clear vision [8–10]. In dry eye patients, reduced tear film stability causes an earlier and quicker disruption of its morphology after blinking. This leads to changes in the optical quality of the tear film, reduced retinal image quality and, as a result, an increase in visual symptoms [11–13]. However, the visual disturbance associated with decreased tear film optical quality in dry eye patients is usually difficult to detect with conventional visual acuity measurements [10, 11].

Efforts have been made to capture the visual fluctuation and/or tear film optical quality dynamics by using successive measurements of visual acuity [14–17], wavefront aberration [18, 19] or double-pass image quality [12, 13, 20, 21]. Functional visual acuity (FVA) allows the detection of visual disturbance related to tear film instability in dry eye patients by measuring the temporal changes of subjective visual acuity [15]. The double-pass method possesses an ability to objectively record serial retinal images and then calculate the objective scatter index (OSI) in intervals of 0.5 s for 20 s [13]. The time course of changes in OSI are considered as the tear film optical quality can fluctuate in patients with DED [12, 13, 20].

Although FVA or OSI analysis alone is sensitive enough to assess tear film stability, the combination of the two will provide more reference for understanding the mechanism of visual disturbance in dry eye patients, both objectively and subjectively. However, FVA and OSI can only be measured separately, due to several uncertainties (tear film variations, blinking effect, pupil size, illumination and accommodation), the direct comparison of the data from two instruments may lead to misinterpreting of the results [22]. To facilitate the direct comparison, we developed a simultaneous real-time analysis system (SRTAS) by adding an additional FVA channel to a commercially available double-pass system.

The purpose of this study is to achieve both subjective and objective assessments of tear film instability in DED simultaneously. We also attempted to evaluate the significance of certain metrics derived from the SRTAS for assessing and quantifying tear film instability in DED from two dimensions: subjective visual performance and objective optical quality.

## Methods

### Participants

This prospective, case-controlled study recruited 57 postgraduate students at the Eye Hospital and School of Ophthalmology and Optometry, Wenzhou Medical University from October 2021 to December 2021. Inclusion criteria were as follows: age ≥ 18 years old; best-corrected visual acuity (BCVA) of 0.0 (logMAR) or better. Exclusion criteria were as follows: presence of any ocular conditions that could increase ocular scatter, such as cataracts, corneal dystrophy, corneal opacity, etc.; previous history of ocular surgery or trauma; history of contact lens wear within the past one month; usage of any topical drugs that affect the tear system within 24 h before the examination (such as artificial tears, and etc.); currently taking any systemic drugs that can affect the tear system (such as Roaccutane, etc.). Participants were divided into two groups: dry eye group (DE group) and normal control group (NC group). For the purpose of this study, DED was defined as Ocular Surface Disease Index (OSDI) score ≥ 13 and non-invasive tear break-up time (NIBUT) < 10 s, in accordance with The Dry Eye Workshop II’s recommendations [23]. Similarly, NC group participants were required to have both an OSDI < 13 and NIBUT ≥ 10 s. Participants who were symptomatic without signs or asymptomatic with signs were excluded. To assess the intraobserver variability of the FVA software, another group of participants without any ocular disease other than refractive error was recruited.

### Custom-developed functional visual acuity software

A custom FVA software was first developed to make it possible and feasible for the integration of FVA module and double-pass system. Modifications have been made with standard FVA design [15, 24] to achieve more personalized assessment with well-trained participants and the major difference between the design of custom-developed and standard FVA software was the introduction of reaction time (Table 1). The reaction time was first measured (with optotype 0.2 logMAR unit larger than BCVA) for each participant to generate the subsequent optotype display time, mean reaction time (mRT) and standard deviation (SD) were calculated for four directions of “Tumbling E”, separately, and the optotype display time was initially set to mRT + 2 SD. The examination distance and time duration can be customized as needed (10 s to 5 min, but 20 s was used in this study). Before carrying out the current study, a Bland–Altman analysis was performed in a pilot study with 60 participants to assess the agreement between the visual acuity measured with custom-developed software and Standard Logarithmic Visual Acuity Chart (Xingkang, Wenzhou, China). The letter-by-letter scoring method (0.02 logMAR per letter identified correctly) [25, 26] was used and good agreement was observed (Bland–Altman analysis found a mean difference of 0.00 with 95% limits of agreement ranged from − 0.06 to + 0.06 logMAR, which was within clinically acceptable limits.

**Table 1:** Modifications of the custom-developed functional visual acuity software

Procedure	Response	Features for next optotype		
		Direction	Optotype size	Display time
Reaction time measurement	Correct/incorrect	Random direction of four: up, down, left, right	Maintain constant, 0.2 logMAR unit larger than best-corrected visual acuity	Sustained display until response
Functional visual acuity measurement	Correct; within mRT + 2SD		Decreased by 0.1 logMAR unit	mRT + 2SD
	Correct; response time was longer than mRT + 2SD		Remained unchanged	mRT + 3SD
	Incorrect; within the set display time		Increased by 0.1 logMAR unit	mRT + 3SD
	No response; within the set display time		Increased by 0.1 logMAR unit	mRT + 3SD

*mRT* = mean reaction time; *SD* = standard deviation

### Design of the simultaneous real-time analysis system

**Fig. 1:**
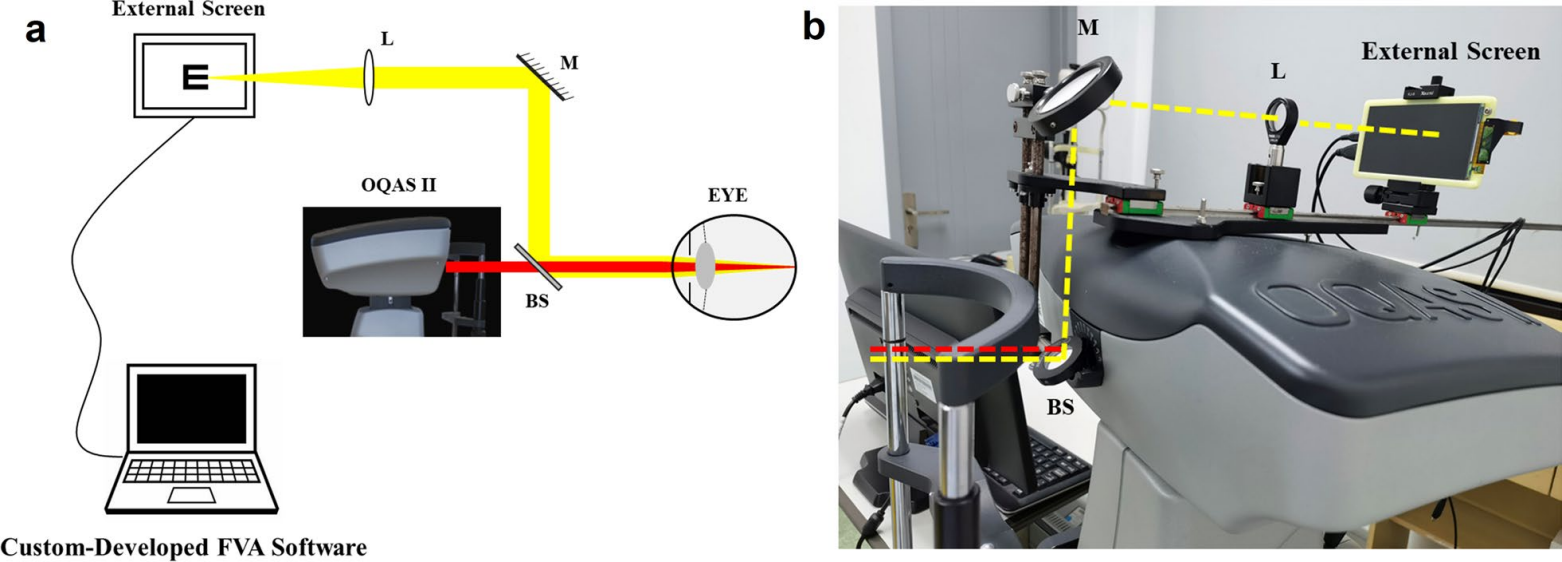
The simultaneous real-time analysis system (SRTAS). **a** Schematic diagram: optical path of the functional visual acuity (FVA) channel is shown in yellow, optical path of Optical Quality Analysis System II (OQAS II) is shown in red (drawing not to scale); **b** A photo of the SRTAS: optical path of the FVA channel is shown in yellow dashed line, optical path of OQAS II is shown in red dashed line. BS, beam splitter; M, mirror; L, lens

The SRTAS consisted of two parts (Fig. 1): Optical Quality Analysis System II (OQAS II; Visiometrics S.L., Tarrasa, Spain) and a FVA channel. The OQAS II was a double-pass system which recorded and analyzed retinal images of a point source. The double-pass retinal images were affected by both intraocular scattering and ocular aberrations, and the analysis of the light distribution of retinal images will provide objective assessment of optical quality which tightly correlated with visual performance. Therefore, the parameters of OQAS II were able to quantify the effect of intraocular scattering and ocular aberrations on visual acuity. Optical quality was quantified using OSI which was defined as the ratio of the intensity at an eccentric location from 12 to 20 min of arc in the double-pass image and the central area of 1 min of arc [27]. The built-in “Tear Film Analysis” function repeatedly measures OSI at intervals of 0.5 s for 20 s.

For the FVA channel, an external screen (size: 121 mm × 68 mm, resolution: 2560 × 1440) was used to display optotypes. A minus lens (L, f =  − 100.0 mm, ACN254-100-A, Thorlabs) provided a minification of 0.4 when was placed 15 cm away from the screen. A beam splitter (M254C45, Thorlabs) and a mirror (M, ME2-P01, Thorlabs) were used on the optical path to separate the two channels. At 45° angle of incidence, the specific beam splitter provided a transmission > 92% from 710 to 1200 nm and a reflection > 97% from 400 to 690 nm. Therefore, there was almost no loss of energy for the double-pass system which used a laser diode with a 780 nm wavelength. The image of optotype was 40 cm in front of the eyes through the entire optical path of the FVA channel.

### Tear function and ocular surface evaluation

The right eye from each participant completed a comprehensive dry eye evaluation in the following order: 1) Clinical interview for ocular health; 2) Subjective refraction; 3) OSDI questionnaire: a trained interviewer (YJM) administered the Mandarin Chinese version of the OSDI questionnaire (Allergan, Inc., Irvine, CA, USA), and the scores were calculated for total and three subscales: vision-related function, ocular symptoms, and environmental triggers, separately [28]; 4) Slit-lamp examination: careful investigation to rule out pathologic conditions other than DED; 5) Keratograph 5 M (K5M; OculusOptikgerate GmbH, Wetzlar, Germany): lower tear meniscus height (LTMH) was measured directly below the pupil center with a built-in caliper function, automated assessment of NIBUT with three measurements was performed and the median value was recorded, all measurements were taken by the same investigator (YJM); 6) Measurements of SRTAS: the BCVA and the “reaction time” through FVA channel were first measured under full refractive correction with trial lenses.

Topical anesthesia (0.5% proparacaine hydrochloride, Alcon, Belgium) was instilled into both eyes 5 min before SRTAS examination to minimize discomfort and prevent reflex tearing and blinking [17, 24]. The participants were instructed to blink twice like normal and then keep their eyes open. Subsequently, the FVA measurement was manually started immediately after the initiation of “Tear Film Analysis” to achieve simultaneous analysis. Three repeated measurements (20 s) of SRTAS under blink suppression condition were performed with a 5-min interval. For each participant, at least two measurements were used in the subsequent analysis and any measurement with blinking or significant head movement was excluded; 7) Corneal fluorescein staining was graded according to the National Eye Institute’s grading scale [29].

### Metrics derived from the simultaneous real-time analysis system

The outcome metrics of the SRTAS were denoted as mean FVA, visual maintenance ratio (VMR), mean OSI, OSI maintenance ratio (OSI-MR), visual acuity break-up time (VA-BUT) and OSI break-up value (OSI-BUV). The mean FVA was defined as the mean value of timewise change of visual acuity during the overall 20 s, and it was obviously affected by the baseline BCVA of each participant. The participant who had significant better baseline static BCVA may present overall better mean FVA regardless of the visual fluctuation. To evaluate and compare the decline of visual acuity in participants with different baseline BCVA, the VMR was calculated as follows [15, 30]: VMR = (lowest logMAR visual acuity − mean FVA) / (lowest logMAR visual acuity − baseline BCVA), the lowest logMAR visual acuity was set at 2.7 for calculation as proposed by previous studies [15, 24, 30]. The VMR can be used to assess the difference between dynamic visual fluctuation and baseline visual acuity. Accordingly, mean OSI and OSI-MR were calculated based on the serial OSI values measured by OQAS II: OSI-MR = (highest OSI value − mean OSI) / (highest OSI value − initial OSI), the initial OSI was the first OSI value during the 20 s. The largest OSI value obtained in the current study of all participants was 7.7, and thus the highest OSI value was set at 10 for calculation. The VA-BUT derived from FVA measurement was defined as the time between blink and the observation of visual acuity first decreased by two lines (0.2 logMAR unit) or more during the overall examination, if no decrease of visual acuity for more than two lines was noted, the VA-BUT was set to 20 s. The OSI-BUV was defined as the OSI value corresponding to the VA-BUT, if VA-BUT was set to 20 s, the OSI-BUV was set to be the last OSI value during the 20 s. According to the definition, VA-BUT was the time interval that elapsed between a complete blink and the appearance of a significant visual deterioration. The clinical relevance of this metric was to assess the overall tear film stability from the perspective of subjective visual performance. Serial visual acuity values in intervals of 0.5 s for 20 s were extracted from the FVA curve using the GetData Graph Digitizer 2.2 (http://getdata-graph-digitizer.com) to achieve a one-to-one correspondence between FVA values and OSI values.

### Statistical analysis

The sample size was estimated using G*Power (version 3.1.9.7, University of Kiel, Germany) [31]. A large effect size (d = 0.868) obtained from a pilot study with 14 participants, 7 in each group, was used and the minimum sample size requirement for an independent samples t-test with an alpha level of 0.05 (two-tailed), a power of 0.8, and an allocation ratio of 2:1, was calculated to be 50 (33 in DE group and 17 in NC group).

All statistical analyses were performed using SPSS (IBM Corp. Released 2017. IBM SPSS Statistics for Windows, Version 25.0. Armonk, NY: IBM Corp). The Shapiro–Wilk’s test was used for testing normality. All continuous variables were expressed as the mean ± standard deviation, and categorical variables were summarized as percentages. Independent samples t-test or Mann–Whitney U test was used for comparison between two groups and the Pearson or Spearman correlation analysis was performed to investigate the correlations between parameters depending on the normality of parameters. Correlation coefficients (absolute value) ranging from 0.70 to 0.90, 0.50 to 0.70, and 0.25 to 0.50 were categorized as high, moderate, and low correlation, respectively. The receiver operating characteristic (ROC) analysis was performed to evaluate and compare the performance of discrimination for parameters. The area under curve (AUC) of the ROC curve was calculated and the optimal cut-off was determined based on the value of the Youden index. The unary linear regression analysis and logarithmic regression analysis were made to determine the correlation between FVA and OSI values. The intraobserver variability of FVA measurements was assessed using the within-subject standard deviation, test–retest repeatability, within-subject coefficient of variation, and intraclass correlation coefficient (ICC). A *P* value less than 0.05 was accepted as statistically significant.

## Results

The DE group included 37 participants (37 right eyes, 5 males and 32 females) and the NC group included 20 participants (20 right eyes, 7 males and 13 females). Table 2 summarizes the clinical features and tear function parameters. There were significant differences in OSDI scores, NIBUT, LTMH, and corneal fluorescein staining scores between the two groups. Twenty-six normal participants (26 right eyes, 9 males and 17 females), aged 25.27 ± 1.46 years old, were enrolled for the intraobserver variability analysis of FVA measurements under the natural blinking condition. The repeatability of mean FVA and VMR, both with three consecutive measurements, was assessed and the results are presented in the Additional file 1: Table S1. The ICC of mean FVA (0.872, 95% CI: 0.772 to 0.936) and VMR (0.737, 95% CI: 0.566 to 0.861) demonstrated moderate to good repeatability, and the within-subject standard deviation of both (0.020, 0.007, respectively) were within clinically acceptable limits.

**Table 2:** Clinical features and tear function parameters in the dry eye and normal control groups

Parameter	Dry eye group (n = 37)	Normal control group (n = 20)	*P* value
Gender (%, female)	86.5	65.0	0.119
Age (years)	24.6 ± 1.5	24.6 ± 0.9	0.784
SE (D)	− 3.22 ± 1.75	− 2.52 ± 2.19*	0.172
BCVA (logMAR)	− 0.03 ± 0.05	− 0.01 ± 0.02	0.073
OSDI total	33.24 ± 13.51	5.25 ± 4.29*	< **0.001**
Ocular symptoms	35.58 ± 13.42*	8.75 ± 6.88	< **0.001**
Vision-related function	30.89 ± 18.23	3.85 ± 5.35	< **0.001**
Environmental triggers	35.81 ± 17.19	4.58 ± 6.88	< **0.001**
NIBUT (s)	6.19 ± 1.53*	17.52 ± 3.63*	< **0.001**
LTMH (mm)	0.15 ± 0.04*	0.23 ± 0.04*	< **0.001**
CFSS	0.38 ± 0.59	0.00 ± 0.00	**0.005**

*SE* = spherical equivalent; *BCVA* = best-corrected visual acuity; *OSDI* = Ocular Surface Disease Index; *NIBUT* = non-invasive tear break-up time; LTMH = lower tear meniscus height; *CFSS* = corneal fluorescein staining score

*Normal distribution, bold font indicates statistical significance

### Comparative analysis of the metrics between groups

The outcome metrics of the SRTAS were calculated for each participant and the means of the metrics were compared. As shown in Table 3, the OSI-MR, VMR and VA-BUT were significantly lower and mean OSI, mean FVA and OSI-BUV were significantly higher in the DE group compared with those of the NC group. This indicated that the participants in the DE group presented worse visual performance and tear film optical quality under blink suppression condition for 20 s.

**Table 3:** Comparative analysis of the metrics derived from the simultaneous real-time analysis system (SRTAS) between the two groups

Parameter	Dry eye group (n = 37)	Normal control group (n = 20)	*P* value
Mean OSI	1.53 ± 0.47*	0.73 ± 0.27*	< **0.001**
OSI-MR	0.92 ± 0.04	0.98 ± 0.01*	< **0.001**
Mean FVA	0.11 ± 0.04*	0.05 ± 0.03*	< **0.001**
VMR	0.95 ± 0.01*	0.98 ± 0.01*	< **0.001**
VA-BUT (s)	8.82 ± 2.25*	14.80 ± 3.82*	< **0.001**
OSI-BUV	1.36 ± 0.53	0.77 ± 0.29*	< **0.001**

*OSI* = objective scatter index; *OSI-MR* = OSI maintenance ratio; *FVA* = functional visual acuity; *VMR* = visual maintenance ratio; *VA-BUT* = visual acuity break-up time; OSI-*BUV* = OSI break-up value

*Normal distribution, bold font indicates statistical significance

### Correlation analyses of the metrics derived from the simultaneous real-time analysis system

The relationships between the FVA and OSI parameters were investigated among all participants (57 eyes). Moderate correlations were noted between mean OSI and FVA-related parameters (mean FVA, VMR, VA-BUT: 0.53, − 0.56, − 0.53, respectively, Pearson, Pearson, Spearman correlation, *P* < 0.01 for all). Moderate to high correlations were noted between OSI-MR and FVA-related parameters (mean FVA, VMR, VA-BUT: − 0.62, 0.71, 0.64, respectively, Spearman correlation and *P* < 0.01 for all). Low correlations were noted between OSI-BUV and FVA-related parameters (mean FVA, VMR, VA-BUT: 0.37, − 0.4, − 0.28, respectively, Spearman correlation for all, *P* < 0.01, *P* < 0.01, *P* < 0.05).

**Fig. 2:**
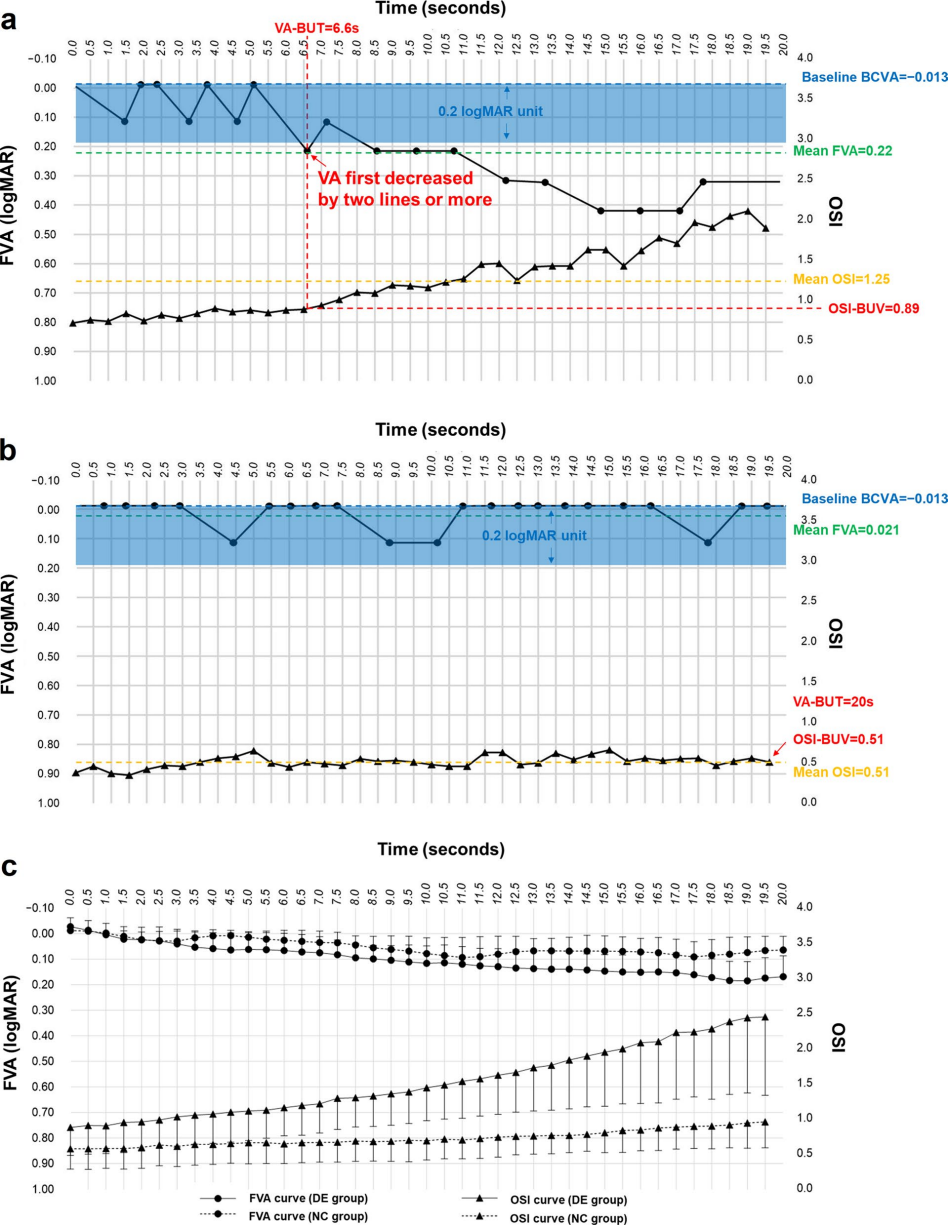
The dual Y-axis charts of both functional visual acuity (FVA) and objective scatter index (OSI) values measured by the simultaneous real-time analysis system. The solid line with dots is the FVA curve, and the solid line with triangles is the OSI curve. **a** A dual Y-axis chart of a participant in the dry eye group, the visual acuity break-up time (VA-BUT) was defined as the time between blink and the observation of visual acuity first decreased by two lines or more (red arrow). The OSI break-up value (OSI-BUV) was defined as the OSI value corresponding to the visual acuity break-up time (VA-BUT) in the dual Y-axis chart. For this participant, the VA-BUT is 6.6 s and the OSI-BUV is 0.89; **b** A dual Y-axis chart of a participant from the normal control group. No decrease of visual acuity for more than two lines was noted. The VA-BUT was set to 20 s and the OSI-BUV was defined as the OSI value of the last measurement. For this participant, the VA-BUT is 20 s and the OSI-BUV is 0.51; **c** The dual Y-axis charts of both FVA and OSI values in intervals of 0.5 s for 20 s. In the DE group, the OSI demonstrated a more pronounced ascending pattern over time than NC group, while the FVA demonstrated a pronounced and corresponding descending pattern. Error bars represent standard deviation. BCVA, best-corrected visual acuity; DE group, dry eye group; NC group, normal control group

The repeated values of both FVA and OSI were plotted in a dual Y-axis chart to carry out further analysis (Fig. 2). Figure 2a and b show the dual Y-axis chart of one participant in each group, and the definitions of VA-BUT and OSI-BUV are illustrated. The repeated visual acuity values in intervals of 0.5 s for 20 s were compared with the corresponding OSI values at each time point (Fig. 2c). A more pronounced ascending pattern of OSI and corresponding descending pattern of FVA were noted in DE group than NC group. To further explore the impact of increasing OSI on FVA outcomes, the FVA was calculated as a function of the OSI values in DE group: FVA = (0.107 × OSI) − 0.064 (R^2^ = 0.88,* P* < 0.001). The logarithmic regression analysis gave better results than the linear regression in terms of goodness-of-fit (R^2^ = 0.94, *P* < 0.001).

### Correlation analyses between patient-reported symptoms and tear film functional indications

Analysis of the relations between patient-reported symptoms (OSDI scores) and various tear film functional indications among all participants revealed that the metrics derived from the SRTAS were moderately correlated with the OSDI scores (Table 4). Among these metrics, the VA-BUT possessed the highest correlation coefficients with OSDI total, OSDI ocular symptoms and OSDI vision-related function (− 0.64, − 0.63, − 0.62, respectively, *P* < 0.01). Both NIBUT and LTMH demonstrated moderate correlations with OSDI scores as well. Only corneal fluorescein staining score was not correlated with the OSDI scores. The correlation analyses revealed that the patient-reported symptoms were associated with increased mean FVA, mean OSI and OSI-BUV (positive correlation), and decreased VMR, VA-BUT and OSI-MR (negative correlation).

**Table 4:** Correlations between subjective symptoms and various tear film functional indications (n = 57)

OSDI scores	Mean FVA	VMR	VA-BUT	Mean OSI	OSI-MR	OSI-BUV	NIBUT	LTMH	CFSS
Total	0.51**	− 0.58**	− 0.64**	0.57**	− 0.59**	0.44**	− 0.63**	− 0.66**	0.20
Ocular symptoms	0.48**	− 0.53**	− 0.63**	0.56**	− 0.54**	0.44**	− 0.64**	− 0.63**	0.21
Vision-related function	0.49**	− 0.60**	− 0.62**	0.55**	− 0.58**	0.40**	− 0.57**	− 0.59**	0.21
Environmental triggers	0.47**	− 0.53**	− 0.55**	0.50**	− 0.56**	0.43**	− 0.63**	− 0.65**	0.17

Spearman correlation for all; **P < 0.01

*OSDI* = Ocular Surface Disease Index; *OSI* = objective scatter index; *OSI-MR* = OSI maintenance ratio; *FVA* = functional visual acuity; *VMR* = visual maintenance ratio; VA-*BUT* = visual acuity break-up time; *OSI-BUV* = OSI break-up value; *NIBUT* = non-invasive tear break-up time; *LTMH* = lower tear meniscus height; *CFSS* = corneal fluorescein staining score

### Discrimination performance of the metrics derived from the simultaneous real-time analysis system

**Fig. 3:**
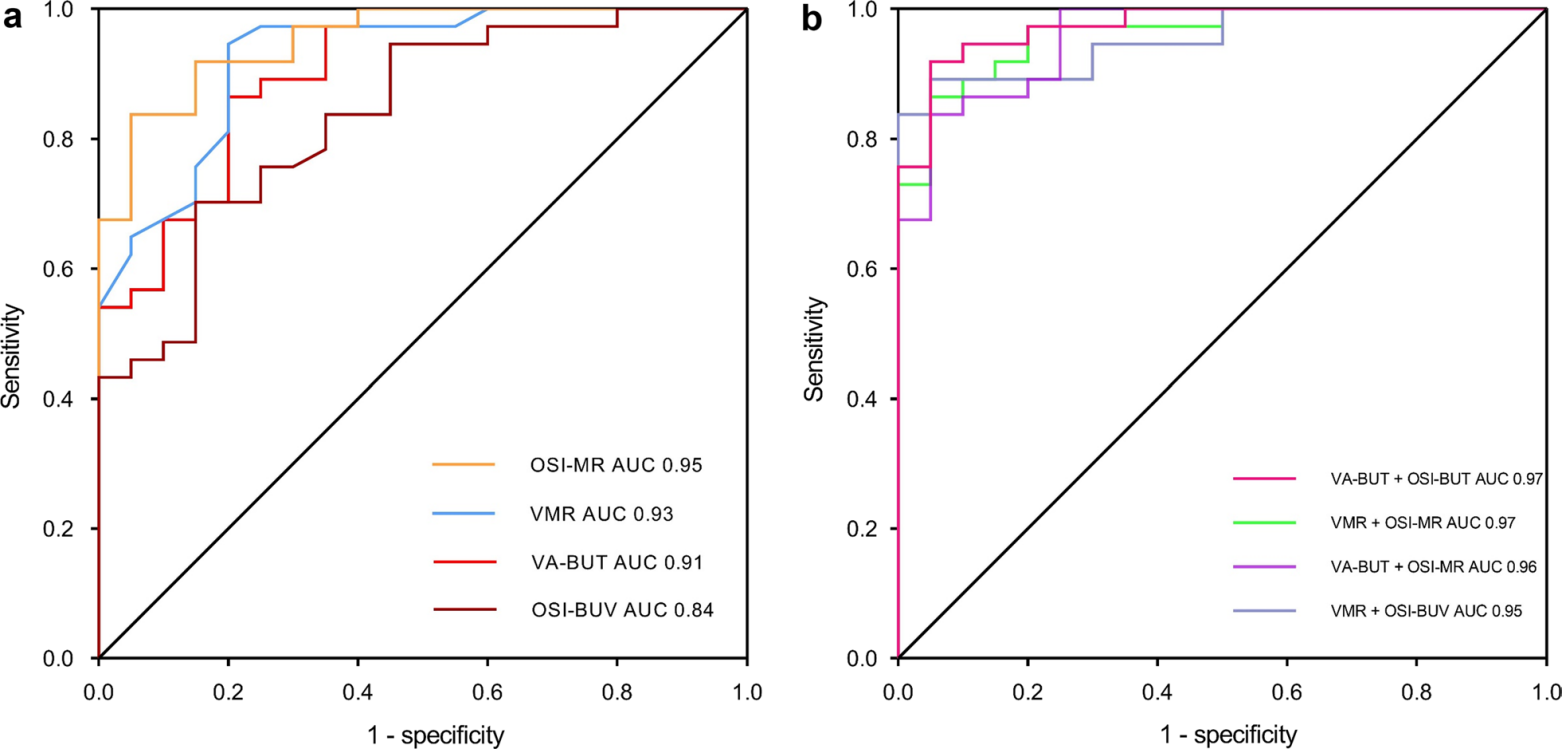
The receiver operating characteristic (ROC) curve using metrics derived from the simultaneous real-time analysis system in the discrimination of eyes with dry eye disease from normal controls.** a** Using each metric alone; **b** Using combined metrics. AUC, area under curve; OSI, objective scatter index; VMR, visual maintenance ratio; OSI-MR, OSI maintenance ratio; VA-BUT, visual acuity break-up time; OSI-BUV, OSI break-up value

Discriminating abilities of the metrics derived from the SRTAS were investigated using ROC analysis. Figure 3 showed the ROC curves of each single metric (Fig. 3a) and combined metrics (Fig. 3b, combination of FVA parameters and OSI parameters). Table 5 shows the AUC and the optimal cut-off with the corresponding sensitivity and specificity for both single and combined metrics used to discriminate eyes with DED from normal controls.

**Table 5:** Discrimination performance of the metrics derived from the simultaneous real-time analysis system in discriminating eyes with dry eye disease from normal controls

Parameter	AUC	Optimal cut-off	Sensitivity (%)	Specificity (%)
OSI-MR	0.95	0.96	95.0	83.8
VMR	0.93	0.97	80.0	94.6
Mean OSI	0.93	1.07	83.8	90.0
VA-BUT (s)	0.91	11.09	80.0	86.5
Mean FVA	0.85	0.076	83.8	80.0
OSI-BUV	0.84	1.06	70.3	85.0
VA-BUT + OSI-BUV	0.97	−	91.9	95.0
VMR + OSI-MR	0.97	−	94.6	90.0
VA-BUT + OSI-MR	0.96	−	83.8	95.0
VMR + OSI-BUV	0.95	−	89.2	95.0

*OSI-MR* = objective scatter index maintenance ratio; *VMR* = visual maintenance ratio; *VA-BUT* = visual acuity break-up time; *OSI-BUV* = OSI break-up value

## Discussion

To our knowledge, this is the first study to incorporate continuous FVA measurements into a tear film optical quality dynamics analysis system which allowed the simultaneous assessment of both subjective visual acuity decline and objective optical quality deterioration during the same tear film break-up cycle in participants diagnosed with DED. Several studies with small sample sizes have successfully acquired simultaneous measurements of both optical quality (wavefront aberrations [32], intraocular scatter [33]) and visual performance (contrast sensitivity [32, 33]) during tear film break-up in subjects wearing contact lenses. Liu et al. [32] developed a three-channel optical system to achieve simultaneous measurements of wavefront aberrations, letter contrast sensitivity, and retro-illumination images. Declines of contrast sensitivity were observed corresponding with the deteriorations of image quality caused by optical degradation of tear film quality during an 18-s blink-suppression period. Their results provided sufficient evidence of a causal relationship between tear film break-up and visual function loss in contact lens wearers. However, the dynamic tracking of contrast sensitivity decline was challenging and may suffer from a significant delay (4.5- or 7.5-s) [32] between the contrast sensitivity decline and the optical quality degradation. Therefore, the accuracy of real-time tracking of visual function loss is reduced. Furthermore, the wearing of contact lenses in these studies introduces additional variability in the results due to the artificial alteration of the tear-film stability [33–36]. In our study, the clinical significance was further enhanced by recruiting a larger sample size of participants diagnosed with DED and the measurements were taken of the “natural tear film” without contact lenses. In terms of time delay, the modified FVA used in our study was also believed to provide better accuracy of real-time monitoring of visual performance compared to contrast sensitivity tracking which has been reported with significant delay.

In clinical practice, the spatial location and area of tear film break-up will demonstrate significant variability from one interblink interval to another even for the same subject [37]. Therefore, the separation of FVA and OSI measurement with two different instruments increases the complexity and uncertainty of establishing a reliable relationship between the subjective visual acuity decline and objective optical quality deterioration. The major advantage of the SRTAS is the combination of subjective visual performance and objective measurement of tear film stability in real-time, enabling a direct comparison of FVA and OSI measurements in the same tear film break-up cycle. During the blink suppression period, a pronounced ascending pattern of OSI and corresponding descending pattern of FVA were observed in DED in the dual Y-axis chart (Fig. 2c). This provided intuitive evidence for the mechanism of visual fluctuation in DED: tear film break-up in the pupillary area caused optical quality degradation (OSI values increased with time), which in turn caused a visual acuity decline (FVA values increased with time). Based on the simultaneous real-time measurements, the impact of increasing OSI on FVA outcomes can be further quantified using linear and logarithmic regression analysis in the DE group. Our results show that the linear and logarithmic regression model achieved a high predicting accuracy and can be simply interpreted as follows: an increase in one unit over the OSI scale led to an increase of FVA of 0.107 logMAR (R^2^ = 0.88, *P* < 0.001), representing about a drop in one line of visual acuity. This quantitative analysis allowed a more in-depth analysis of the real-time visual performance prediction for different tear film optical quality values. The moderate to high correlations between mean OSI, OSI-MR and FVA-related parameters (mean FVA, VMR and VA-BUT) have not been reported previously. These findings of the current study indicate that the tear film optical quality metrics (OSI and OSI-MR) can provide objective and quantitative reference of the subjective visual performance during tear film break-up in the pupillary area.

In previous studies [12, 13, 20], tear film optical quality has been assessed as temporal changes of OSI in subjects with DED. Increasing OSI over time was considered a common feature of tear film instability in DED and was also observed in this study. Furthermore, the OSI-MR was calculated based on the serial OSI values measured in a span of 20 s. This enabled the comparison of the OSI variations in participants with different baseline ocular scatter. Among OSI-related parameters, the OSI-MR showed the highest correlations with mean FVA, VMR and VA-BUT (− 0.62, 0.71, 0.64, respectively, *P* < 0.01). This indicates that the OSI-MR is superior to other OSI-related parameters in quantifying visual acuity decline caused by tear film optical quality changes. From a mathematical point of view, the calculation of OSI-MR which incorporated the influence of baseline OSI was able to provide a quantitative assessment of how well the optical quality of tear film can be maintained in a break-up cycle. This evaluation index will apparently be more representative for the OSI variations by assessing the difference between dynamic OSI changes and baseline OSI.

The concept and connotation of FVA were first introduced by Goto et al. [16] as a simulation of vision with unconscious blink suppression for daily activities. It was initially measured manually with sustained eye opening for 10 to 20 s with the aid of topical anesthesia. Since then, the FVA was considered a favorable method to detect visual disturbance related to tear film instability in DED [14, 17, 30, 38–40] and different methodologies of FVA testing were proposed (automatic measurement [17, 30], shortened measurement time [41], under natural blinking condition [42]). The key features of the modified FVA testing methodology in our study were as follows: introduction of “reaction time” to personalize the optotype display time; blink suppression for 20 s with topical anesthesia, rather than 30 or 60 s in Kaido et al.’s studies [14, 39, 42]. The accuracy and effectiveness of this modified FVA testing were investigated and our findings showed promising results: the mean FVA, VMR and VA-BUT were significantly different between the DE and NC groups, and all parameters showed high discrimination abilities to discriminate eyes with DED from normal controls (AUC 0.85, 0.93, 0.91, respectively); the FVA-related parameters also demonstrated significant correlations with patient-reported symptoms (OSDI scores). Unlike our study, Kaido et al. reported there was no statistically significant differences in FVA parameters (mean FVA and VMR) under blink suppression condition between the dry eye subjects and normal controls [42], and the effectiveness of discriminating between DED and normal controls using each FVA parameter was low (AUC: mean FVA 0.525, VMR 0.553) [14]. The discrepancy of results can be attributed to different FVA testing methodologies utilized and diverse study population recruited across studies. In Kaido et al.’s studies, FVA was measured either under natural blinking condition for 60 s [14, 42] or under blink suppression condition with topical anesthesia for 30 s [42]. Spontaneous blinking, which facilitated the distribution and the formation of the tear film, was believed to be essential for maintaining a good optical quality of the ocular surface [42–44]. Therefore, natural blinking will providing a better visual performance during the FVA measurements. This may decrease the discrimination abilities of FVA parameters since certain subjects with tear film instability can also maintain good visual acuity by blinking in a timely manner throughout the test. On the contrary, blink suppression for an adequate and appropriate period will bring about visual acuity decline associated with optical quality deterioration due to tear film break-up in DED. However, tear film optical quality deterioration can also be noticed with an interblink interval longer than 10 s in normal subjects [45] and excessively prolonged time of blink suppression will significantly increase tear film irregularity in all subjects which may in turn bridge the gap of tear film stability between DED and normal controls. This could partially explain the discrepancy that the difference in FVA parameters between groups was significant for 20 s of blink suppression in our study and was not significant for 30 s (considered as excessively prolonged time) of blink suppression [42]. Moreover, there were differences in the clinical features of normal controls (sampling bias) between studies. In our study, normal controls were defined as OSDI score < 13 and NIBUT ≥ 10 s, however, in Kaido et al.’s studies [14, 42], normal controls included some subjects with short tear film break-up time, but no symptoms (average tear film break-up time was 6.6 ± 3.3 s and 5.9 ± 3.0 s in control groups), which may reduce the essential difference of the tear film stability between DED and normal controls in their studies. The FVA parameters in our study was expected to produce higher sensitivities and specificities in discriminating definite normal controls from DED [23].

The discordance between measured signs and patient-reported symptoms of DED has been observed [46–49], and this poor correlation can be partially explained by relatively poor repeatability of objective signs, subjective nature of symptoms, and individual variations in cognitive responses to ocular symptoms [50–52]. Interestingly, we found moderate correlations between the metrics derived from the SRTAS and the OSDI scores (Table 4). This suggested that these metrics (mean FVA, VMR, VA-BUT, mean OSI, OSI-MR, OSI-BUV), which directly quantified the optical quality deterioration and visual disturbance caused by tear film instability, may potentially be favorable indicators for evaluating patient-reported symptoms in DED. Among these metrics, the VA-BUT demonstrated the strongest correlations with OSDI total, OSDI ocular symptoms and OSDI vision-related function (− 0.64, − 0.63, − 0.63, respectively, *P* < 0.01). This was expected considering the implication of VA-BUT which represented a vision-related, symptom-based, quantifiable, and subjective cut-off for visual acuity decline. However, the significant correlations between the signs and symptoms in this study should be interpreted with consideration of the sample-specific characteristics in mind. The participants recruited in this study consisted of healthy younger adults only, eliminating several factors that may contribute to the discrepancies between the signs and symptoms of DED: age [46, 53], individual cognitive responses (postgraduate students) [50, 51] and the ability to cooperate with the test. Additional studies are needed to determine if our findings can be replicated among different sample populations.

Based on the significant differences of the metrics derived from the SRTAS between the two groups, it was reasonable to investigate the performance of each metric in discriminating eyes with DED from normal controls. Here, our non-overlapping setting of the DE group (OSDI score ≥ 13 and NIBUT < 10 s) and NC group (OSDI score < 13 and NIBUT ≥ 10 s) can produce remarkably high sensitivity and specificity [23]. The OSI-MR alone appeared to exhibit the best performance of the six metrics for the detection of DED, achieving an AUC of 0.95 with sensitivity of 95.0% and specificity of 83.8%. Even the lowest AUC of OSI-BUV reached 0.84 with a sensitivity of 70.3% and specificity of 85.0%, which was acceptable as a diagnostic metric for DED. Parallel testing of both FVA and OSI will potentially increase sensitivity and specificity by adding extra information from a different dimension (i.e., the addition of subjective visual acuity decline to objective optical quality deterioration) [23], and this synergistic effect could be most dramatic under the simultaneous real-time condition. Furthermore, the combinations of FVA parameters and OSI parameters in this study were valid and can further improve the discriminating abilities of SRTAS metrics with the highest AUC value to be 0.97 (VA-BUT + OSI-BUV, sensitivity of 91.9% and specificity of 95.0%). The potential significance of these metrics for diagnosing DED has been well explored in the current sample population. However, poorer sensitivity and specificity could be expected in a real-world population which includes uncategorized or preclinical subjects, especially subjects with inconsistent signs and symptoms.

A major limitation is that the sampling bias explained previously may restrict the generalization of study findings and the true performance of these diagnostic metrics are expected to be compromised in the general population. However, the promising findings with the current sample population will provide certain references or inspirations for eye care practitioners or researchers in this field and could serve as an encouraging starting point for our future research. It will be exciting to see the performance of these metrics in the real-world clinical setting. In addition, previous studies [14, 42] had recommended that the measurement of FVA should be performed under natural blinking condition without topical anesthesia for 60 s. In tandem, our testing duration of SRTAS has been limited by the built-in “Tear Film Analysis” function of OQAS II and was set to 20 s. The measurement of SRTAS was performed under blink suppression condition, therefore, the optimal testing duration and the influence of different blink conditions have not yet been explored. Although the repeatability of FVA measurements was assessed in a group of normal participants and the moderate to good repeatability allowed us to sufficiently interpret the main findings of the current study, the intra- and inter-observer variability of the simultaneous measurement with the current prototype of SRTAS was not assessed and should be further investigated.

## Conclusions

Simultaneous real-time measurement of the subjective visual acuity decline and objective optical quality deterioration can facilitate and deepen the understanding of the interrelationship between tear film instability and visual performance as well as the underlying mechanism of visual fluctuation in DED. Although the measurements were carried out simultaneously, the significance of these metrics derived from the SRTAS can be appreciated separately: the OSI-related metrics can provide objective and quantitative reference that are correlated with both subjective visual performance and patient-reported symptoms, supporting these metrics as potential indicators for assessing and diagnosing DED. The FVA-related metrics based on a modified testing methodology were believed to be vision-related, symptom-based, and quantifiable for evaluating visual acuity decline which was associated with tear film instability in DED.

## Supplementary Information

**Additional file 1:** **Table S1.** Intraobserver repeatability of functional visual acuity measurements in normal participants (26 eyes).

## Data Availability

The datasets used and/or analyzed are available from the corresponding author upon reasonable request.

## Abbreviations

DED: Dry eye disease
FVA: Functional visual acuity
OSI: Objective scatter index
SRTAS: Simultaneous real-time analysis system
BCVA: Best-corrected visual acuity
DE group: Dry eye group
NC group: Normal control group
OSDI: Ocular Surface Disease Index
NIBUT: Non-invasive tear break-up time
mRT: Mean reaction time
SD: Standard deviation
OQAS II: Optical Quality Analysis System II
LTMH: Lower tear meniscus height
VMR: Visual maintenance ratio
OSI-MR: OSI maintenance ratio
VA-BUT: Visual acuity break-up time
OSI-BUV: OSI break-up value
ROC: Receiver operating characteristic
AUC: Area under curve
ICC: Intraclass correlation coefficient
