# Supplementary material for: Simultaneous real-time analysis of tear film optical quality dynamics and functional visual acuity in dry eye disease
Source: Eye Vis (Lond). 2023 Apr 2;10:16. doi: 10.1186/s40662-023-00333-6 (PMC10068140; doi:10.1186/s40662-023-00333-6)
Supplement: Supplementary file 1 — Additional file 1: Table S1. Intraobserver repeatability of functional visual acuity measurements in normal participants (26 eyes). [file 40662_2023_333_MOESM1_ESM.docx]

**Additional file 1:** **Table S1.** Intraobserver repeatability of functional visual acuity measurements in normal participants (26 eyes).

| Parameter | Mean±SD | S_w_ | TRT | CoV (%) | ICC (95% CI) |
| --- | --- | --- | --- | --- | --- |
| Mean FVA | 0.03±0.05 | 0.020 | 0.056 | 58.65 | 0.872 (0.772 to 0.936) |
| VMR | 0.97±0.01 | 0.007 | 0.020 | 0.75 | 0.737 (0.566 to 0.861) |

FVA = functional visual acuity; VMR = visual maintenance ratio; SD = standard deviation; S_w_ = within-subject standard deviation; TRT = test-retest repeatability; CoV = within-subject coefficient of variation; ICC = intraclass correlation coefficient
